# Editorial: Use of teledentistry and artificial intelligence to enhance dental care for vulnerable populations

**DOI:** 10.3389/fdmed.2025.1723059

**Published:** 2025-11-03

**Authors:** David O. Danesh, Walter Yu Hang Lam, Duangporn Duangthip

**Affiliations:** ^1^ College of Dentistry, The Ohio State University, Columbus, OH, United States; ^2^ Department of Dentistry, Nationwide Children’s Hospital, Columbus, OH, United States; ^3^ Faculty of Dentistry, The University of Hong Kong, Sai Ying Pun, Hong Kong SAR, China

**Keywords:** teledentistry, artificial intelligence (AI), remote patient monitoring (RPM), mobile health (mHealth), vulnerable populations (health), dental care

**Editorial on the Research Topic**
Use of teledentistry and artificial intelligence to enhance dental care for vulnerable populations

Improving access to dental care for vulnerable populations is essential to achieving health equity. These groups—such as low-income individuals and those in underserved areas—face persistent barriers including cost, transportation, language, and systemic bias. Addressing these gaps requires community engagement, coordinated research and the integration of advanced technology that prioritize equity and cultural competence. Innovation continues to transform dental practice with technologies like teledentistry and artificial intelligence (AI) offering promising solutions to enhance care delivery and accessibility. Teledentistry serves as an important tool for vulnerable populations with applications such as screening and diagnosis, provision of oral health education, and engagement of patients and caregivers through digital platforms which can increase access to care in underserved areas (1–3). AI in dentistry can be utilized for AI-driven diagnostics and treatment planning, personalized dental care programs, and development of AI-based educational tools (4–6). Combined approaches can include hybrid models integrating teledentistry and AI for mobile health (mHealth) and remote patient monitoring, as well as research and development of new technologies for dental care (7, 8).

The Research Topic on this research topic “*Use of Teledentistry and Artificial Intelligence to Enhance Dental Care for Vulnerable Populations*,” presents diverse perspectives on the use of emerging technologies to improve oral health, covering the mobile app interventions, ethical considerations in AI use, and the quality of AI-generated dental advice. Four research articles that offer new insight were included. One original research article in this collection used the Telesmile mobile app to improve oral health for populations with special health care needs. A perspective article examined ethical considerations for using AI among vulnerable populations. A mini review outlined innovations on the horizon for emerging technologies, including teledentistry, artificial intelligence, and hybrid models. Finally, another original research article examined the quality of artificial intelligence-generated pediatric dental advice across different platforms.

An article by Fageeh et al. utilized a mobile app (Telesmile) to enhancing oral health and oral hygiene practices among the blind and deaf populations in Jazan Province in Saudi Arabia. The Telesmile mobile application, which included audio technique and video demonstration, can significantly enhance oral hygiene knowledge among blind and deaf people compared to regular oral hygiene instructions. The perspective article by McGrath et al. highlighted digital health technologies in remote oral health monitoring and management, such as artificial networks, augmented reality/virtual reality, data mining, digital biomarkers, mobile health, chatbots, and wearables, to enhance diagnosis, treatment planning, and disease prediction. The authors discussed concerning issues among vulnerable populations, including obtaining consent, privacy and security issues, considerations with electronic health records, and accessibility and inclusivity. Drafta et al. synthesize 21 studies in a narrative review that outline digital technologies in dentistry including teledentistry, artificial care, and hybrid models. The authors describe implications for patient satisfaction, acceptability, barriers, and facilitators to care, as well as the horizon for future innovations. The last original research article by Kapoor et al. compared the quality of advice across three artificial intelligence platforms in regard to clinical accuracy, clarity, completeness, relevance, and absence of misleading information.

The articles in this collection share developments in teledentistry, artificial intelligence, and other technologies will allow clinicians, researchers, and patients to revolutionize how dentistry is practiced worldwide and to ensure oral health for vulnerable populations. Together, these innovations have the potential to reduce oral health disparities, enhance preventive care, and support more equitable healthcare delivery.
